# Minocycline mitigates sepsis‐induced neuroinflammation and promotes recovery in male mice: Insights into neuroprotection and inflammatory modulation

**DOI:** 10.14814/phy2.70032

**Published:** 2024-10-06

**Authors:** Mahmoud Hosseini, Zahra Bardaghi, Hedyeh Askarpour, Arezoo Rajabian, Maryam Mahmoudabady, Sadegh Shabab, Zahra Samadi‐Noshahr, Hossein Salmani

**Affiliations:** ^1^ Psychiatry and Behavioral Sciences Research Center Mashhad University of Medical Sciences Mashhad Iran; ^2^ Applied Biomedical Research Center Mashhad University of Medical Sciences Mashhad Iran; ^3^ Department of Developmental Biology Washington University School of Medicine St. Louis Missouri USA; ^4^ Bioenvironmental Health Hazards Research Center Jiroft University of Medical Sciences Jiroft Iran; ^5^ Neuroscience Research Center Mashhad University of Medical Sciences Mashhad Iran; ^6^ Chabahar Faculty of Medical Sciences, School of Medicine Iranshahr University of Medical Sciences Chabahar Iran; ^7^ Department of Physiology and Pharmacology, Faculty of Medicine Sabzevar University of Medical Sciences Mashhad Iran

**Keywords:** brain inflammation, minocycline, oxidative stress, sepsis

## Abstract

Sepsis is associated with brain injury and acute brain inflammation, which can potentially transition into chronic inflammation, triggering a cascade of inflammatory responses that may lead to neurological disorders. Minocycline, recognized for its potent anti‐inflammatory properties, was investigated in this study for its protective effects against sepsis‐induced brain injury. Adult male C57 mice pretreated with minocycline (12.5, 25, and 50 mg/kg) 3 days before sepsis induction. An intraperitoneal injection of 5 mg/kg LPS was used to induce sepsis. Spontaneous locomotor activity (SLA) and weight changes were assessed over several days post‐sepsis to monitor the recovery of the mice. The expression of inflammatory mediators and oxidative stress markers was assessed 24 h post sepsis. Septic mice exhibited significant weight loss and impaired SLA. Initially, minocycline did not attenuate the severity of weight loss (1 day) or SLA (4 h post‐sepsis), but it significantly accelerated the recovery of the mice in later days. Minocycline dose‐dependently mitigated sepsis‐induced brain inflammation and oxidative stress. Our findings demonstrate that pretreatment with minocycline has the potential to prevent brain tissue damage and accelerate recovery from sepsis in mice, suggesting that minocycline may serve as a promising therapeutic intervention to protect against sepsis‐induced neurological complications.

## 1 INTRODUCTION

Sepsis‐associated encephalopathy (SAE) is a frequently encountered complication of sepsis, characterized by a spectrum of cognitive impairments ranging from confusion, disorientation, and impaired consciousness to agitation, delirium, and, in severe cases, coma (Tauber et al., 2021). It is estimated that a substantial proportion, up to 70%, of septic patients experience varying degrees of neurological dysfunction (Gofton & Young, 2012). While the precise mechanisms underpinning SAE remain elusive, mounting evidence suggests several potential contributing factors, including microglial activation, neuroinflammation, oxidative and nitrosative stress, excitotoxicity, and blood–brain barrier disruption (Tauber et al., 2021; Widmann & Heneka, 2014).

Beyond the acute phase, sepsis survivors often experience a chronic phase of SAE, characterized by persistent cognitive impairment, increased incidence of psychiatric disorders such as anxiety and depression (Chung et al., 2020), and a diminished quality of life (Korosec Jagodic et al., 2006). Studies have revealed that sepsis survivors are at risk of developing enduring brain dysfunctions and may even be predisposed to neurological disorders such as Alzheimer's and Parkinson's diseases in the future (Chung et al., 2020). Furthermore, investigations utilizing animal models of sepsis have consistently demonstrated that sepsis may cause long‐lasting neuroinflammation and behavioral abnormalities (Bardaghi et al., 2023; Zhao et al., 2020). In a previous study, our research team observed that mice subjected to sepsis displayed heightened sensitivity to secondary inflammations, exacerbating their behavioral responses to systemic LPS injections compared to control mice (Salmani et al., 2022). Thus, the transition from acute to chronic brain inflammation following sepsis has garnered significant research attention in recent years, as preventing such a transition holds promise for averting the long‐term consequences of sepsis.

Minocycline, a semi‐synthetic tetracycline derivative, is well known for its potent anti‐inflammatory and neuroprotective properties (Garrido‐Mesa et al., 2013). It has exhibited efficacy in a range of neuroinflammatory and neurodegenerative conditions, largely attributed to its capacity for suppressing microglial activation (Yrjänheikki et al., 1999), reduce oxidative stress (Amirahmadi et al., 2022; Kraus et al., 2005), prevent apoptosis (He et al., 2021), inhibit iNOS activation (Chen et al., 2000), and modulate inflammatory mediators within the central nervous system (CNS) (Kielian et al., 2007; Yrjänheikki et al., 1999). We have shown in our prior study that minocycline protected the brain against the chronic brain inflammation and long‐term consequences of sepsis in mice model of sepsis (Hosseini et al., 2024). Minocycline is a highly lipophilic molecule that can penetrate the CNS and exert direct effects within the CNS (Garrido‐Mesa et al., 2013). These properties make minocycline an attractive candidate for mitigating sepsis‐induced brain injury. Here, we hypothesized that minocycline can prevent brain inflammation during sepsis and prevent deleterious effects of sepsis in the brain. This study aimed to investigate the potential effects of minocycline against the acute effects of sepsis on the brain and the course of mice recovery from the sepsis. This research may contribute to developing novel strategies for managing and mitigating sepsis‐induced brain injury, with potential implications for improving patient outcomes and quality of life.

## 2 MATERIALS AND METHODS

### 2.1 Animals

Male C57 mice (8–10 weeks old, 25–30 g) were used in the study. Before the experiment, mice were acclimated to a new housing condition for 1 week, and they maintained in standard condition in a temperature of 22 ± 2°C, a 12/12‐h light/dark cycle, and provided with ad libitum access to standard rodent chow (Javaneh Khorasan Co. Mashhad, Iran) and water. Additionally, the mice were habituated to daily handling by the experimenters during the acclimation period.

### 2.2 Study design

**FIGURE 1:**
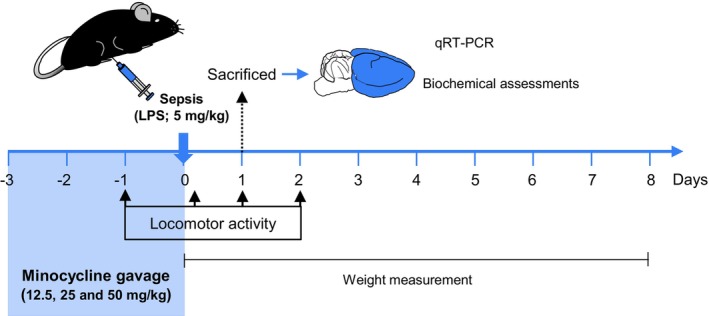
Experimental Design. Mice were subjected to a single intraperitoneal injection of LPS (5 mg/kg) to induce sepsis. Minocycline was administered via gavage once daily. The assessment of locomotor activity and monitoring of weight changes were conducted over the course of several days following sepsis induction. The expression of inflammatory mediators and the measurement of oxidative stress markers were performed 24 h after sepsis in brain tissue.

Eighty‐five mice were randomly divided in Control, Sepsis, Sepsis/Mino 12.5, Sepsis/Mino25 and Sepsis/Mino50 groups (*n* = 17 mouse/group). The sample size was chosen based on prior studies based on the primary outcome being mice revery from sepsis (Bardaghi et al., 2023; Salmani et al., 2022). A single mouse was considered as an experimental unit. Inclusion criteria was the age and the weight of mouse and exclusion criteria was dying of mice during the experiment. Mice received intraperitoneal injection of bacterial lipopolysaccharide (LPS from *E. coli* O55:B5, Sigma‐Aldrich, Product NO: L2880) in 5 mg/kg dose dissolved in 0.2 mL saline or an equivalent volume of saline (in the control group) to induce sepsis (Anderson et al., 2015; Salmani et al., 2022; Zhao et al., 2020). Mice were kept in warm and quiet place and monitored for water and food availability. Eight hours after sepsis induction mice received subcutaneous injection of 0.3 mL saline to prevent dehydration. None of the mice died due to sepsis induction. Minocycline (Sigma‐Aldrich, Cat No. M9511) was administered via oral gavage at three distinct doses (12.5, 25, and 50 mg/kg) once daily, starting 3 days before LPS injection and continuing until the day of sepsis induction (Hoshino et al., 2017; Yang et al., 2022). The control and sepsis groups received the same volume of the vehicle (10% DMSO). Oral gavage was chosen because it mimics the human route of drug delivery, thereby providing results that are more directly translatable to clinical settings. Baseline weights were recorded before sepsis induction, and changes in body weight were monitored for 8 days post‐sepsis. A cohort of mice (5 mouse/per group) was euthanized 24 h after sepsis induction to assess inflammatory mediators in the brain and oxidative stress markers. For this purpose, the mice were anesthetized via i.p. injection of ketamine (80 mg/kg) and xylazine (12.5 mg/kg), sacrificed, and their brains were collected. A separate cohort (12 per group) was designated for behavioral studies and monitored for weight and spontaneous locomotor activity (Figure 1).

### 2.3 Behavioral tests

#### 2.3.1 Spontaneous locomotor activity

To assess the recovery of mice from sepsis, their spontaneous locomotor activity was evaluated in an open field area (a polypropylene cage measuring 38 × 24 × 20 cm) at 4, 24, and 48 h after LPS injection. The area was divided into six 12 × 12 cm squares (Figure 3), and line crossing and rearing behavior were recorded during a 3‐minute test (Bardaghi et al., 2023; Murray et al., 2013). The experimenter counting the mice behavior was blinded to the animal grouping. To eliminate olfactory cues, a 10% ethanol solution was used to clean the cage after each assessment.

### 2.4 Tissue collection

The mice were anesthetized with a ketamine/xylazine injection (80 and 12.5 mg/kg, respectively) and then sacrificed. Subsequently, the brain tissues were swiftly removed, and the cortex and hippocampus were carefully dissected. The hippocampal tissues were preserved in RNAlater (Yekta Tajhiz Azma, Cat No. YT9085) for the evaluation of mRNA expression, while the cortex was stored at −80°C for biochemical assessments.

### 2.5 RNA isolation and RT‐PCR

Total RNA was extracted from hippocampal tissues using the Favorgen Biotech RNA extraction kit (Cat. No. FATRE 96001). The quality and quantity of the extracted RNA were assessed through agarose gel electrophoresis and UV spectrophotometry (NanoDrop 1000TM, USA), respectively. Subsequently, cDNAs were synthesized from the extracted RNA using the easy cDNA synthesis kit (Cat No. A101161, Parstous, Iran). For quantitative real‐time PCR (qRT‐PCR) analysis, the synthesized cDNAs were combined with custom‐designed and optimized primers specific for GAPDH, IL‐1β, TNF‐α, and NF‐κB (as listed in Table 1). The qRT‐PCR procedure was carried out using RealQ Plus 2X‐MasterMix Green (Cat No. A325402, Amplicon, Denmark). To determine relative mRNA expression levels, normalization was performed with GAPDH using the 2^−ΔΔCt^ method.

**TABLE 1:** The sequence of primers.

Gene	Primers (5′ → 3′)	Accession number
GAPDH	Forward: CAACGACCCCTTCATTGACC Reverse: CTTCCCATTCTCGGCCTTGA	NM_001411843.1
NF‐κB	Forward: CCAAGGACATGACTGCTCAA Reverse: AGACGCTGCCTCTGAAGTTT	NM_001177370.1
IL‐1β	Forward: GACTTCACCATGGAATCCGT Reverse: TGCTCATTCACGAAAAGGGA	NM_008361.4
TNF‐α	Forward: AGGCTGTCGCTACATCACTG Reverse: CTCTCAATGACCCGTAGGGC	NM_001278601.1

### 2.6 Biochemical assessment

For all the biochemical test the experimenter conducting the tests was unaware of animal grouping.

#### 2.6.1 Measuring MDA levels, SOD activity, and total thiol content

Thiobarbituric acid‐reactive‐substances (TBARS) assay was used to measure Malondialdehyde (MDA) levels. Briefly, tissue homogenates were mixed with the reagent (40% trichloroacetic acid, 5 M HCl, and 2% TBA) and heated in boiling water for 15 min. Then, the samples were centrifuged (1500 × *g*, 10 min), and the absorbance of the supernatant was read at 535 nm (Salmani et al., 2020).

For thiol content measurement, Ellman's method was used. Briefly, Ellman's reagent (2, 2′‐dinitro‐5, 5′‐dithiodibenzoic acid (DTNB, Sigma Aldrich Company, USA, Cat. No. 103291) DTNB) was mixed with tissue homogenates and incubated at room temperature for 20 min to develop a yellow color. Subsequently, the absorbance was measured at 412 nm (Samadi‐Noshahr et al., 2021).

Madesh and Balasubramanian method was used to measure superoxide dismutase (SOD) activity. In brief, cortex homogenates were incubated with pyrogallol and MTT (3‐(4, 5‐dimethylthiazol‐2‐yl) 2, 5‐diphenyltetrazolium bromide), (Sigma Aldrich Company, USA, Cat. No. 475989). The reaction was stopped by adding DMSO, and the absorbance was read at 420 nm (Eshaghi Ghalibaf et al., 2023).

#### 2.6.2 AChE activity

AChE (Acetylcholinesterase) activity was determined using Ellman's method in the cortical tissues (Hosseini et al., 2015). Briefly, biological samples were incubated with acetylthiocholine (acetylthiocholine iodide [Tokyo Chemical Industry Co., Japan, Product Number: A0116]) and DTNB to initiate the enzymatic reaction, and the absorbance was measured at 412 nm.

### 2.7 Statistical analyses

Data analysis and graph preparation were performed using GraphPad Prism (version 9.5.1 for Windows, GraphPad Software, San Diego, California, USA, www.graphpad.com). The normality of data distribution was assessed using the Shapiro–Wilk test, while the homogeneity of variances was examined with Bartlett's test. The ROUT method with a *Q*‐coefficient of 1% was applied to identify potential outliers.

In cases where homogeneity of variances was not met, data were subjected to Welch's ANOVA test, followed by unpaired *t*‐tests with Welch's correction for further analysis. Otherwise, data were analyzed using ordinary one‐way ANOVA, followed by Tukey's or Dunnett's multiple comparisons test. Graphs present data as mean ± SD.

A two‐way repeated measure ANOVA (Time × Treatment) with Geisser–Greenhouse's correction was employed for weight and spontaneous locomotor activity data, followed by multiple comparisons using Bonferroni correction. Statistical significance was determined at *p* < 0.05.

## 3 RESULTS

### 3.1 Weight changes

The percentage changes in body weight were measured over 8 days following sepsis induction. A two‐way repeated‐measures ANOVA revealed significant main effects of time (*F* (2.878, 158.3) = 161.4, *p* < 0.001) and treatment (*F* (4, 55) = 6.924, *p* < 0.001) and an interaction between time × treatment (*F* (28, 385) = 6.149, *p* < 0.001).

**FIGURE 2:**
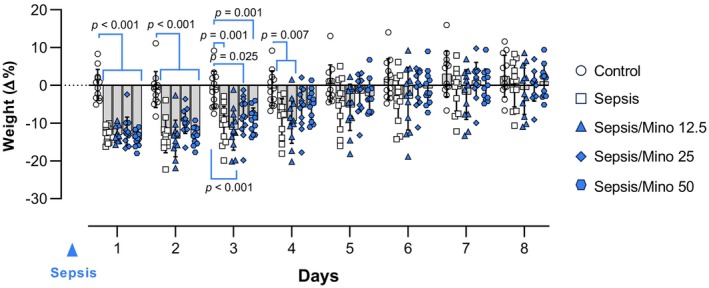
Daily weight changes post sepsis induction. Data were analyzed using two‐way ANOVA and Bonferroni multiple comparison test and presented as mean ± SD (*n* = 12). Each individual data points shows the data of a single mouse.

In all mice injected with LPS, including those pretreated with minocycline, a severe reduction in body weight (up to 13%) was observed at 24 h post‐sepsis induction (*p* < 0.001 vs. control group). By day 4 post‐sepsis induction, mice treated with minocycline at doses of 25 and 50 mg/kg had regained a significant portion of their lost weight (*p* = 0.230 and *p* = 0.192, respectively, compared to the control group), while the Sepsis and Sepsis/Mino12.5 groups still had significantly lower weights than the control group (*p* = 0.007 and *p* = 0.007, respectively). Subsequently, all septic mice, including those treated with minocycline, recovered the weight they had lost, and by the end of the 8th‐day post‐sepsis, all groups had reached the same weight as the control mice (Figure 2).

### 3.2 Spontaneous locomotor activity

Locomotor activity in an open field area was monitored at 4, 24, and 48 h after sepsis induction. Rearing behavior, which serves as an indicator of exploratory activity and can decrease due to sickness behavior, was also measured to assess the mice's recovery from sepsis. Significant differences in the main effects of time (rearing: *F* (2.422, 133.2) = 266.3, *p* < 0.001; crossing: *F* (2.521, 138.6) = 296.4, *p* < 0.001), treatment (rearing: *F* (4, 55) = 53.58, *p* < 0.001; crossing: *F* (4, 55) = 49.43, *p* < 0.001), and they interaction (rearing: *F* (12, 165) = 15.05, *p* < 0.001; crossing: *F* (12, 165) = 15.00, *p* < 0.001) were found.

**FIGURE 3:**
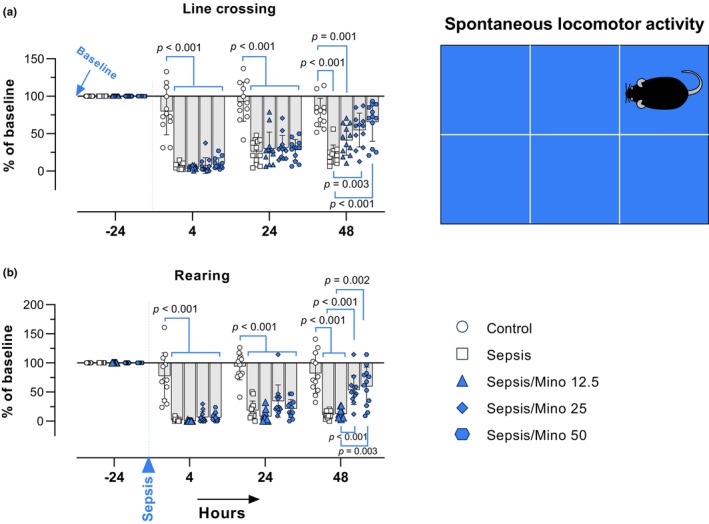
Spontaneous Locomotor activity. Spontaneous Locomotor Activity. (a) Percentage changes in line crossings and (b) percentage changes in rearing behavior during the 3‐minute test period. Data were analyzed using a two‐way repeated‐measures ANOVA, followed by the Bonferroni multiple comparison test, and are presented as mean ± SD (*n* = 12). Each individual data point represents the data of a single mouse.

Both rearing behavior (*p* < 0.001) and line crossing (*p* < 0.001) significantly decreased (up to 90%) in septic mice 4 h post‐sepsis, and minocycline did not prevent or ameliorate this effect (rearing, *p* < 0.001; crossing, *p* < 0.001 Minocycline groups vs Control). At 24 h, all septic mice, regardless of receiving minocycline treatment or the vehicle, exhibited a significant decrease in rearing and crossing (*p* < 0.001 vs control). However, at 48 h, mice treated with minocycline at doses of 25 and 50 mg/kg showed an increase in line crossing compared to the vehicle‐treated septic mice (*p* = 0.003, *p* < 0.001, respectively). Similarly, rearing behavior increased at these doses (*p* < 0.001 and *p* = 0.002, respectively) compared to the vehicle‐treated septic mice (Figure 3a,b). Overall, these results indicate that pretreatment with minocycline accelerated the recovery time in post‐septic mice.

### 3.3 The expression of inflammatory mediators

**FIGURE 4:**
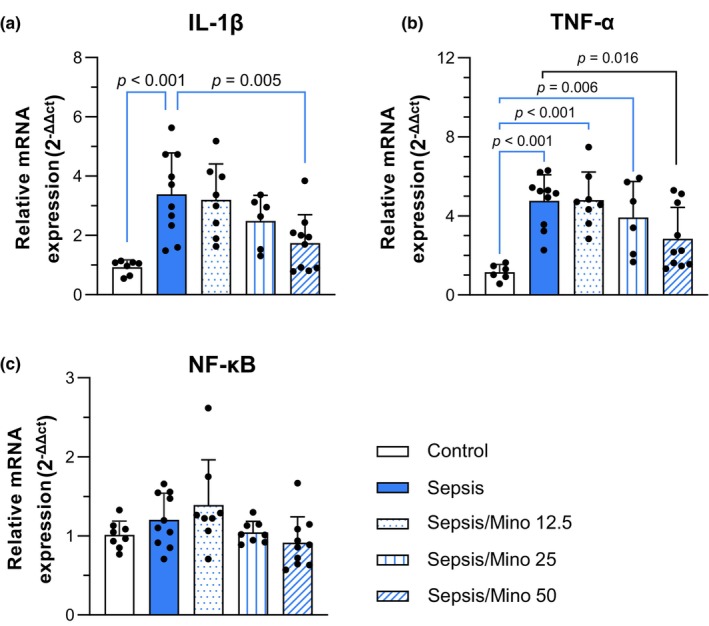
Expression of inflammatory mediators in the hippocampal tissue. mRNA expression of (a) IL‐1β, (b) TNF‐α, and (c) NF‐κB. Each sample was assayed in duplicate (*n* = 6–10). Each sample was assayed in duplicate (*n* = 6–10), with two samples collected from each mouse. Each individual data point represents one sample. Statistical analysis was performed using one‐way ANOVA with Dunnett's multiple comparisons test, and the results are presented as mean ± SD.

The mRNA expression of IL‐1β in the hippocampal tissues significantly differed between groups (*F* (4, 36) = 7.639, *p* < 0.001). Dunnett's multiple comparisons test showed that sepsis significantly increased IL‐1β expression in the hippocampal tissues of septic mice (*p* < 0.001). Minocycline at 50 mg/kg dose significantly reduced IL‐1β expression (*p* = 0.005) (Figure 4a).

TNF‐α mRNA expression showed significant differences among groups (*F* (4, 35) = 8.432, *p* < 0.001). Septic mice, including Sepsis, Sepsis/Mino 12.5, and Sepsis/Mino 25, showed a 4‐to‐5‐fold increase in the TNF‐α expression compared to control mice (*p* < 0.001, *p* < 0.001, *p* = 0.006). Minocycline at 50 mg/kg significantly reduced TNF‐α expression compared to the Sepsis group (*p* = 0.016) (Figure 4b).

One‐way ANOVA analysis showed no significant differences in the NF‐κB expression in the hippocampal tissues (*F* (4, 39) = 2.560, *p* = 0.054) (Figure 4c).

### 3.4 Biochemical assessments

#### 3.4.1 Lipid peroxidation

**FIGURE 5:**
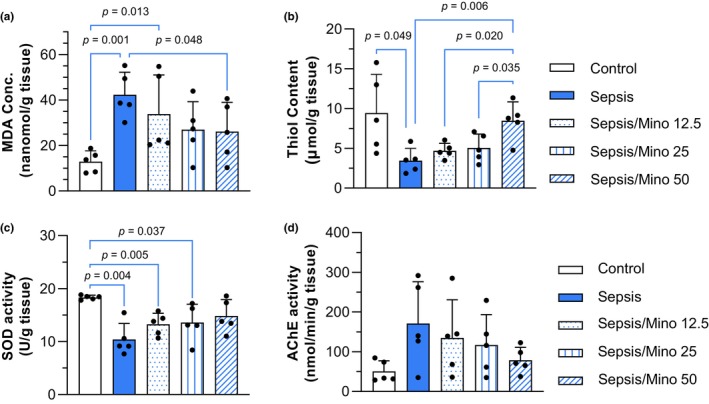
Biochemical assessments in the cortical tissues 24 h post‐sepsis. (a) MDA concentration, (b) Total thiol content (*n* = 5), (c) SOD activity, (d) AChE activity. Data were subjected to one‐way ANOVA with Tukey's post hoc test for statistical analysis and are presented as mean ± SD (*n* = 5). Each individual data point shows the data of a single mouse.

Treatment had a significant effect on the MDA levels (*F* (4, 20) = 3.998, *p* = 0.015). The LSD post hoc test demonstrated a significant increase in MDA levels in the cortical tissues of septic mice compared to the control group (*p* = 0.001). Among the minocycline‐treated groups, a significant reduction in MDA levels was observed in the 50 mg/kg dose (*p* = 0.048), whereas there were no significant changes in the 12.5 and 25 mg/kg groups (*p* = 0.283 and *p* = 0.060, respectively) (Figure 5a).

#### 3.4.2 Thiol content

Welch's ANOVA test revealed significant differences in thiol content among cortical tissues (*W* (4.000, 9.518) = 4.179, *p* = 0.032). Unpaired t‐tests with Welch's correction indicated a significant reduction in thiol content in the cortical tissues of the Sepsis group (*p* = 0.049). However, treatment with Minocycline at a dose of 50 mg/kg prevented this reduction in thiol content (*p* = 0.0058). In both the Sepsis/Mino 12.5 and Sepsis/Mino 25 groups, thiol content was significantly lower than in the Sepsis/Mino 50 group (*p* = 0.02, *p* = 0.035, respectively) (Figure 5b).

#### 3.4.3 SOD activity

Significant differences were detected between the groups using Welch's ANOVA test (*W* (4.000, 8.313) = 14.99, *p* < 0.001). Unpaired t‐tests with Welch's correction revealed a significant reduction in SOD activity in the Sepsis group (*p* = 0.004), as well as in the Sepsis/Mino 12.5 (*p* = 0.005) and Sepsis/Mino 25 (*p* = 0.037) groups compared to the control group. No significant differences were observed between the sepsis group and minocycline‐treated septic mice (Figure 5c).

### 3.5 AChE activity

One‐way ANOVA showed no significant differences in the AChE activity among groups (*F* (4, 20) = 2.002, *p* = 0.133), indicating that sepsis did not cause a significant change in AChE activity in 24 h post sepsis induction (Figure 5d).

## 4 DISCUSSION

This study evaluated the preventative effects of minocycline against the acute effects of sepsis on the mouse brain. Findings showed that pretreatment of septic mice with minocycline led to a dose‐dependent acceleration of recovery from sepsis and amelioration of inflammatory gene expression and oxidative stress‐related brain injury. The beneficial effects of minocycline against acute and chronic brain injuries have been reported in previous studies in various models. In the cecal ligation and puncture (CLP) model of sepsis, minocycline has been shown to prevent oxidative stress, production of inflammatory cytokines (Michels et al., 2015), long‐term potentiation impairment (Hoshino et al., 2017), and blood–brain barrier permeability (Yang et al., 2022). Moreover, the neuroprotective effects of minocycline against traumatic brain injury (Lu et al., 2022), epilepsy‐induced brain inflammation (Wang et al., 2015), and ischemic stroke‐induced brain inflammation and injury (Quezya et al., 2020) have been previously reported.

Consistent with earlier reports, severe weight loss and locomotor activity occurred in septic mice, a severe systemic response typically associated with septic conditions (Bardaghi et al., 2023; Salmani et al., 2022). Initially, minocycline pretreatment did not prevent these responses, resulting in minocycline‐treated mice displaying a comparable degree of locomotor reduction and weight loss. It is not surprising since the same degree of response had been reported low and high doses of LPS. For example, Zhao et al. (2020) reported severe systemic responses and inflammation in mice injected with both 1 and 5 mg/kg LPS. Interestingly, the lower LPS dose allowed for recovery from the systemic inflammatory response, while the higher dose led to the development of chronic brain inflammation lasting over 10 months (Zhao et al., 2020). Thus, while minocycline significantly reduced inflammatory gene expression and mitigated oxidative stress damage during the initial phase of sepsis, it could not reduce behavioral response of mice to sepsis.

Moreover, our findings showed that AChE activity was not significantly increased in the acute phase of sepsis. However, in the same model of sepsis we have previously shown that sepsis significantly increases AChE activity one‐month after sepsis induction along with significant cognitive impairment (Bardaghi et al., 2023; Hosseini et al., 2024).

Microglial cells, the brain's resident immune cells, play a crucial role in the brain's response to inflammation. Following injury, resting microglia become activated and shift to the M1 phenotype, producing various cytotoxic factors such as superoxide, nitric oxide, IL‐1β, and TNF‐α. Subsequently, microglia transition to the M2 phenotype, contributing to repair, regeneration, and immunoregulation (Cherry et al., 2014). However, in post‐septic animals induced by LPS injection, studies have shown chronic activation of microglial cells and prolonged neuroinflammation (Anderson et al., 2015; Qin et al., 2007). In the CLP model, M1 and M2 phenotype markers coexistence has also been reported early after sepsis induction up to 30 days (Michels et al., 2020). Our recent research further supported these findings, indicating that post‐septic mice exhibit an exacerbated immune response to secondary inflammatory insults, marked by the overexpression of inflammatory mediators within the brain and heightened behavioral responses, thus suggesting fundamental changes in microglial properties in the post‐septic brain (Salmani et al., 2022). IL‐1β and TNF‐α are the important inflammatory cytokines produced by immune cells in response to inflammatory insult or injury (Rahmani et al., 2022). Shortly after systemic inflammation the production of inflammatory mediators including, IL‐1β and TNF‐α raises within the brain tissue, primarily originating from activated microglial cells (Sankowski et al., 2015). In this study, we measured the expression of these two cytokines to assess whether minocycline treatment effectively reduced the severity of neuroinflammation in the brain. We showed that pretreatment of septic mice with minocycline reduced IL‐1β and TNF‐α expression at 24 h post‐sepsis induction. While this reduction in inflammatory mediators did not ameliorate the initial systemic response to sepsis, it did accelerate recovery. This aligns with previous findings suggesting that the course of inflammation during sepsis is closely linked to mature IL‐1β levels in the brain. Elevated IL‐1β levels can contribute to sustained and long‐lasting brain inflammation. Notably, Zhao et al. (2020) demonstrated that while IL‐1R1 deficiency in septic mice does not prevent acute neuroinflammation, it does prevent the transition to chronic brain inflammation. The role of mature IL‐1β levels is crucial in this transition from acute to chronic inflammation during sepsis (Zhao et al., 2020). However, since we did not measure the protein levels of IL‐1β and TNF‐α in this study, further investigation is needed to fully understand the implications of our findings. In the CLP model of sepsis, single intracerebroventricular administration of minocycline has been shown to reduce M1 microglial markers expression early after sepsis induction in the hippocampus (Michels et al., 2020) and inflammatory markers in the hypothalamus (da Costa et al., 2021).

Furthermore, our results revealed that sepsis increased lipid peroxidation and reduced the brain's antioxidant defense system. These findings are consistent with earlier reports indicating systemic inflammation induces oxidative stress in brain tissue (Salmani et al., 2021; Sankowski et al., 2015). In the case of sepsis, those rats who endured CLP showed increased lipid peroxidation within the hippocampus in 10 days after surgery (Comim et al., 2011). It the LPS‐induced sepsis increased MDA and NO levels, and reduced anti‐oxidant capacity has been reported as early as 2 h post sepsis induction (Abd El‐Gawad & Khalifa, 2001). Minocycline significantly prevented oxidative stress in the brain 24 h post‐sepsis induction, indicating minocycline's ability to prevent brain injury during sepsis. Protective effects of minocycline against oxidative stress injury have been previously reported in brain tissue (Amirahmadi et al., 2022). Minocycline has been shown to possess direct radical scavenging properties in the brain cell culture and reduce lipid peroxidation (Morimoto et al., 2005). Being known for its anti‐inflammatory effects and modulating effects on microglial activity (Kim & Suh, 2009), minocycline also prevents oxidative stress arising from an inflammatory response. However, it is worth noting that this study did not assess the effects of minocycline on the long‐term consequences of sepsis, which stands as a limitation warranting further investigation.

## 5 LIMITATIONS

The primary limitation of this study was the exclusive use of the LPS model to induce sepsis, chosen for its consistent recovery time, reproducibility, and manageable inflammatory response. However, it is important to acknowledge that LPS‐induced sepsis may not fully replicate the complexity of human sepsis (Rittirsch et al., 2007). Therefore, future research should consider utilizing other sepsis models, such as the CLP model, to provide a more comprehensive understanding of protective effects of minocycline in sepsis. Second, we only used male mice in this study to reduce the number of variables and thus minimize the number of animals required. However, this may limit the generalizability of our findings.

## 6 CONCLUSION

In conclusion, our findings support the notion that pretreatment with minocycline exerts a significant preventive effect against brain oxidative damage, mitigates neuroinflammation, and accelerates the recovery of mice from the septic condition. Additionally, this study highlights the potential therapeutic value of minocycline in safeguarding neurological well‐being in the context of sepsis, offering promising avenues for future research and clinical applications.

## FUNDING INFORMATION

The Research Deputy of Jiroft University of Medical Sciences financially supported this work.

## CONFLICT OF INTEREST STATEMENT

The authors declare no conflicts of interest.

## ETHICS STATEMENT

This study followed the “Guideline for the Care and Use of Laboratory Animals in Iran” for the experiment on animals. The study was approved in the Research Ethics Committees of Laboratory Animals‐ Jiroft University of Medical Sciences (ID: IR.JMU.AEC.1401.005).

## Supporting information


Data S1:

## Data Availability

The data that support the findings of this study are available from the corresponding author (H. S.) upon reasonable request.
