# Supplementary material for: Minocycline mitigates sepsis‐induced neuroinflammation and promotes recovery in male mice: Insights into neuroprotection and inflammatory modulation
Source: Physiol Rep. 2024 Oct 6;12(19):e70032. doi: 10.14814/phy2.70032 (PMC11456363; doi:10.14814/phy2.70032)
Supplement: Supplementary file 1 — Data S1: [file PHY2-12-e70032-s001.zip › PHYSREP-2024-06-415-s01.docx]

**Dr. Josephine C. Adams**

Editor-in-Chief

***Physiological Reports***

Date: August 11, 2024

Dear **Dr. Adams:**

We are pleased to inform you that we have submitted our revised paper (**PHYSREP-2024-06-415**) entitled " **Minocycline Mitigates Sepsis-Induced Neuroinflammation and Promotes Recovery in Mice: Insights into Neuroprotection and Inflammatory Modulation** " to the **Physiological Reports** journal.

Thank you so much for considering our manuscript. I look forward to hearing from you.

Sincerely,

Hossein Salmani, PhD

Sabzevar University of Medical Sciences

Email: [h.salmani610@ut.ac.ir](mailto:h.salmani610@ut.ac.ir)

[h.salmani610@gmail.com](mailto:h.salmani610@gmail.com)
