# Supplementary material for: Minocycline mitigates sepsis‐induced neuroinflammation and promotes recovery in male mice: Insights into neuroprotection and inflammatory modulation
Source: Physiol Rep. 2024 Oct 6;12(19):e70032. doi: 10.14814/phy2.70032 (PMC11456363; doi:10.14814/phy2.70032)
Supplement: Supplementary file 1 — Data S1: [file PHY2-12-e70032-s001.zip › PHYSREP-2024-06-415-s02.docx]

**Losartan improved hippocampal long-term potentiation impairment induced by repeated LPS injection in rats**

Hossein Salmani, Zahra Bardaghi, Hedyeh Askarpour, Arezoo Rajabian, Maryam Mahmoudabady, Sadegh Shabab, Zahra Samadi-Noshahr, Mahmoud Hosseini*

This study explores the neuroprotective effects of minocycline in sepsis-induced brain injury in mice. Minocycline treatment reduced inflammatory markers, oxidative stress, and enhanced behavioral recovery following sepsis, highlighting its potential as a therapeutic option for preventing neurological complications in septic patients.
