# Supplementary material for: Minocycline mitigates sepsis‐induced neuroinflammation and promotes recovery in male mice: Insights into neuroprotection and inflammatory modulation
Source: Physiol Rep. 2024 Oct 6;12(19):e70032. doi: 10.14814/phy2.70032 (PMC11456363; doi:10.14814/phy2.70032)
Supplement: Supplementary file 1 — Data S1: [file PHY2-12-e70032-s001.zip › PHYSREP-2024-06-415-s03.pdf]

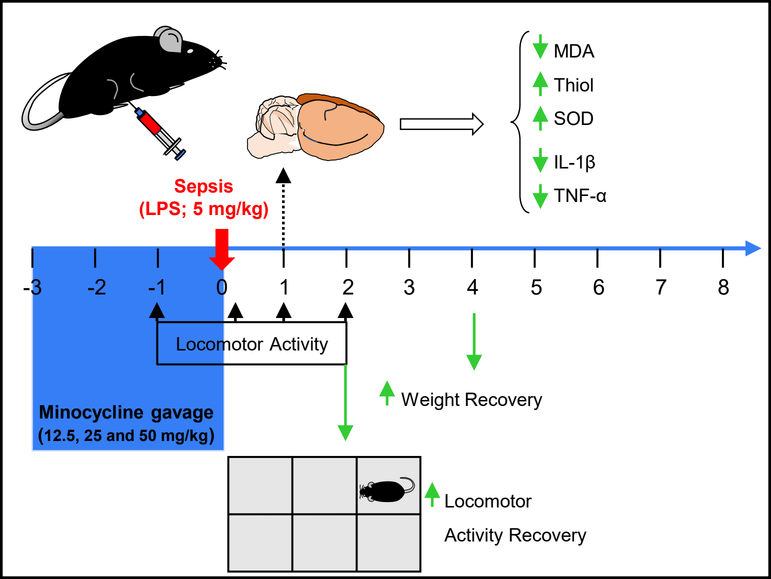
MDA
Thiol
SOD
IL-1β
TNF-α
Sepsis
(LPS; 5 mg/kg)
-3
-2
-1
0
1
2
3
4
5
6
7
8
Locomotor Activity
Weight Recovery
Minocycline gavage
(12.5, 25 and 50 mg/kg)
Locomotor
Activity Recovery
